# A Method of Wound Treatment by the Introduction of Living Cultures of a Spore-Bearing Anaerobe of the Proteolytic Group

**Published:** 1917-11

**Authors:** 


***A Method of Wound Treatment by the Introduction of Living Cultures of a Spore-Bearing Anaerobe of the Proteolytic Group.*** By Robert DONALDSON, Bactereological Specialist, War Hospitals, Reading; Pathologist, Royal Birks Hospital, and J. Leonard JOYCE, Major, R. A. M. C. (T.); Surgical Registrar Royal Birks Hospital, Reading. Abstracted from the *Lancet*, Sept. 22, 1917.

In experimenting with the salt pack treatment of wounds, D. and J. have observed that only those cases make progress in which a foul odor is present. This fact has led them to discover the presence in such cases of a proteolytic anaerobic bacillus (" Reading bacillus ") resembling that of malignant edema but non-pathogenic to man. This appears always when the salt pack treatment is *successful*. It acts, as D. and J. think, in virtue of its proteolytic powers only on devitalized tissue, and possibly upon tox-albumins, and it appears to possess no power of attacking healthy structures.

Since one of the chief factors in keeping a wound septic is the presence of microscopically devitalized tissues, which surgical treatment may fail to remove, the " Reading bacillus ", as D. and J. believe, promises to play an important part in the treatment of wounds. They believe also that the presence of this bacillus and no property of the salt pack itself has been responsible for the success of this treatment. In fact, the substitution of sphagnum moss sown with living cultures of the bacillus has effected cures where other methods including the salt pack have failed.

The authors recommend that cases treated by salt packs or other packing, be inoculated with cultures of this bacillus. They state that in all such cases the wounds must be thoroughly open, exposing every pocket and sinus, so that the packing may completely fill the wound. The surface is then sown with a broth culture of the bacilli, the packing applied, covered with gauze and a cotton pad, and left in position for about seven days. The appearance of the foul odor and the fall in temperature about the third day indicate a favorable reaction in the wound. Several cases are presented. The authors believe this treatment an effective means of removing the menace of devitalized tissues. They expect to make a later communication.

